# Hospital trains on different tracks

**DOI:** 10.2471/BLT.20.020120

**Published:** 2020-01-01

## Abstract

Hospital trains bring health care to people who might otherwise never receive it but connecting them with the broader health system can be a challenge. Sophie Cousins reports.

In 1997, when the United Kingdom of Great Britain and Northern Ireland returned control of Hong Kong to China, each of the 31 provinces on the mainland gave Hong Kong a present.

Nellie Fong, a Hong Kong-based legislator and health-care activist, was part of a committee established to handle the transfer of sovereignty and felt that Hong Kong should reciprocate. The problem was she couldn’t think of 31 meaningful presents. In the end she decided on one: a hospital train.

“I knew about a hospital train in India and I suggested that something similar would make a perfect gift,” she says. “Being mobile, it could benefit many provinces and there were some important health issues it could address.”

The suggestion was accepted and Fong went to India with a group of advisors to see how the train worked.

Called the Lifeline Express, the train had been running since 1991, having been established by the Impact India Foundation (a charitable initiative focused on the prevention and treatment of avoidable disability) in collaboration with Indian Railways, and the Ministry of Health and Family Welfare.

The Lifeline Express had just three carriages and a remit to provide, free of charge, diagnosis and treatment of disabilities caused by polio, which was still endemic in India at that time.

“When we started running the train, we found so many children with polio-related deformities,” says Dr Rohini Chowgule, a trustee at the Impact India Foundation, and formerly head of the medicine department at the Bombay Hospital Institute of Medical Sciences in Mumbai.

But as India intensified its national polio immunization campaign in 1995 and was certified polio-free in 2014, the number of cases declined, and the train was repurposed to address other disabilities including cataracts, cleft lips and hearing problems.

Impressed by the Lifeline Express’s capacity to deliver health care to people in rural and remote areas in India, Fong set up a foundation and invited the Hong Kong business community to fund a similar train in China, focusing solely on cataract surgery.

The first rainbow-painted ‘Eye Train’ – as the trains have come to be known in China – was launched in 1997, providing free cataract surgery. One more was added in 1999, in 2002 and in 2009. Each of the four trains has four carriages with two operating rooms, and accommodation for 12 staff, including doctors, nurses, a technician and administrative staff.

“Patients […] come and stand on a platform in the middle of the night to get treatment.”

Mamta Singh.

The decision to focus on cataract surgery was deliberate as it is a relatively simple procedure that can be rapidly delivered. In 2014, Lifeline Express China expanded into providing free screening for diabetic retinopathy.

According to Fong the trains currently visit three locations each per year, staying at each location for three months, delivering an average 1000 operations per stop. “In just over two decades, Lifeline Express China has provided free cataract surgery to almost a quarter of a million patients,” she says.

Over that same period, Lifeline Express India has taken a different route. The train’s 12-person staff (now running an eight-carriage train and stopping in each location for 21 days), still delivers a range of procedures to address disability, including cataract surgery, and plastic surgery for cleft lip, but since 2009, has also been offering epilepsy diagnosis and treatment on one weekend in each location, and since May 2019 has been offering oral, cervical and breast cancer screening and diagnosis.

However, both epilepsy and cancers require protracted treatment and monitoring, neither of which can be provided by the train’s staff during its 21-day stop. So how do the doctors manage?

According to Chowgule, 858 patients have received a diagnosis since 2009, 318 of which were for cancer. Patients requiring treatment are given the address of the nearest of 200 cancer centres affiliated with the National Cancer Grid. The staff contact the centres and give them the details of the referred patient.

“The train’s cancer department team also follows up with the patient for almost 3 months to ensure that the patient is taking treatment and that he or she is on the right treatment,” Chowgule says.

Despite these efforts, Chowgule acknowledges accessing care is a challenge for some patients, since the cancer grid facilities are under no obligation to provide services free of charge. The National Health Authority, responsible for implementing India’s national health insurance scheme – the Ayushman Bharat programme – and the National Cancer Grid have begun discussions regarding cancer treatment packages and pricing of services to be covered under the programme.

With regard to epilepsy, post-diagnosis follow-up is also challenging. “Epilepsy is a chronic condition and starting treatment is just the beginning,” says Professor Mamta Singh, who, once a month, after a busy week in the neurology department of the All India Institute of Medical Sciences (AIIMS) in Delhi, boards the Lifeline Express with her team to see as many epilepsy patients as possible over the weekend before returning to work on Monday morning.

“The places that the Lifeline Express visits have very few doctors. So, although we educate patients and explain that they need to periodically meet a doctor, I am not sure how many of them are able to do that,” Singh says.

She is in no doubt, however, regarding the importance of the work. On a recent mission to Jharkhand, a state in the east of the country, she saw 230 patients in one town. “The desperation of patients who come and stand on a platform in the middle of the night to get treatment is heart-breaking,” she says.

A study published in the *European Journal of Epilepsy *in February 2019 showed that 87% (581/669) of the AIIMS epilepsy patients were still taking medication five years after diagnosis compared to 72% (351/491) of the Lifeline Express patients.

“The results could have been better, but I was quite happy with what we are being able to achieve even with one visit,” Singh says.

Lack of follow-up is also a concern with supposedly ‘simpler’ procedures, such as cataract surgery. “There’s something of a misperception that the management of cataract requires a once-off intervention,” says Stuart Keel, a disability expert working at the World Health Organization (WHO) in Geneva. “In fact, post-surgical follow up is required to monitor patient progress and intervene where necessary, for example through the provision of laser treatment or glasses to correct changes in vision.”

“… [trains] highlight the shortcomings of the health systems in which they operate.”

However, Keel is willing to acknowledge the contribution hospital trains can make. “Hospital trains, like other forms of mobile health service delivery, can play an important role in providing access to eye care for hard-to-reach populations, but it is important that there is an emphasis on the quality of care provided and that a sustainable infrastructure is available for post-operative services.” he says.

Nellie Fong saw that the trains were not going to be sufficient on their own. Part of the problem was throughput. “We quickly realized that we could only treat around 10 000 people a year,” she says, “So we decided to start training local doctors to do basic surgeries and sending these doctors to local hospitals to run an eye centre, which we also funded.” Another part of the problem was the trains’ inability to handle more complex cases. “Those cases are now referred to the eye centres, which can handle complex cases and train young ophthalmologists,” Fong says.

According to Fong there are now more than 80 eye centres established within existing medical facilities, usually secondary or tertiary referral centres, scattered across China.

China and India are not the only countries running hospital trains. Phelophepa trains in South Africa provide general health, dental and eye checks, visiting up to 70 remote rural communities annually. Two trains traverse the Russian Federation, one of which is the Saint Lukas train, which travels through the vast regions of Krasnoyarsk and Khakassia for 10 months every year.

None of these trains originated as government-funded programmes, all were established as philanthropic or corporate initiatives. Often, they highlight the shortcomings of the health systems in which they operate, rolling into stations where people have limited or no access health care. In some cases, the trains appear only once a year. That one visit is certainly not enough. But, as Professor Mamta Singh points out, it is a visit that can make a big difference.

**Figure:**
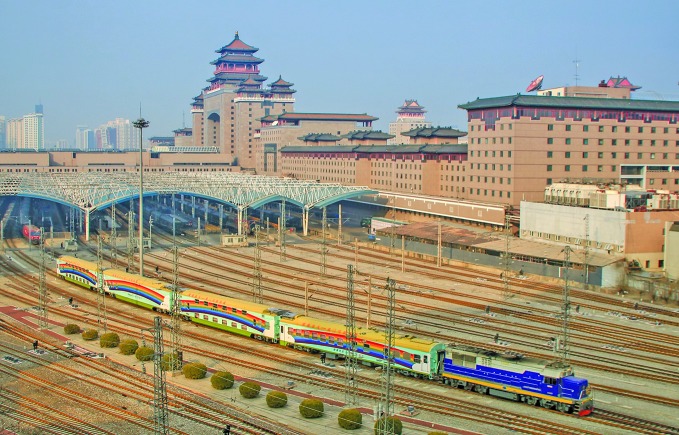
Lifeline Express China

Lifeline Express Foundation

**Figure:**
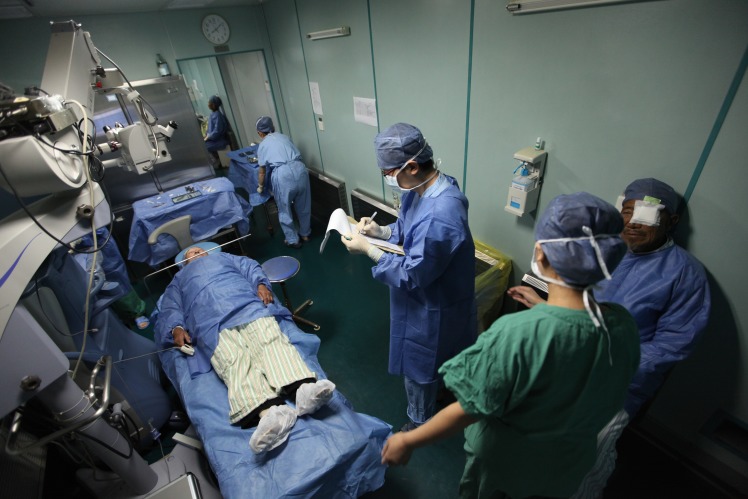
Lifeline Express China doctors perform cataract surgery on the train

Lifeline Express Foundation

